# Elucidating the Binding Mechanism of a Novel Silica-Binding Peptide

**DOI:** 10.3390/biom10010004

**Published:** 2019-12-18

**Authors:** Rachit Bansal, Zehra Elgundi, Andrew Care, Sophia C. Goodchild, Megan S. Lord, Alison Rodger, Anwar Sunna

**Affiliations:** 1 Department of Molecular Sciences, Macquarie University, Sydney NSW 2109, Australia; rachit.bansal@hdr.mq.edu.au (R.B.); andrew.care@mq.edu.au (A.C.); sophie.goodchild@mq.edu.au (S.C.G.); alison.rodger@mq.edu.au (A.R.); 2 ARC Centre of Excellence for Nanoscale Biophotonics, Macquarie University, Sydney NSW 2109, Australia; 3 Graduate School of Biomedical Engineering, University of New South Wales, Sydney NSW 2052, Australia; z.elgundi@unsw.edu.au (Z.E.); m.lord@unsw.edu.au (M.S.L.); 4 Biomolecular Discovery and Design Research Centre, Macquarie University, Sydney NSW 2109, Australia

**Keywords:** solid-binding peptides (SBPs), linker-protein G (LPG), surface plasmon resonance (SPR), quartz crystal microbalance with dissipation monitoring (QCM-D), circular dichroism (CD) spectrometry, equilibrium dissociation constant (*K_D_*)

## Abstract

Linker-protein G (LPG) is a bifunctional fusion protein composed of a solid-binding peptide (SBP, referred as the “linker”) with high affinity to silica-based compounds and a *Streptococcus* protein G (PG), which binds antibodies. The binding mechanisms of LPG to silica-based materials was studied using different biophysical techniques and compared to that of PG without the linker. LPG displayed high binding affinity to a silica surface (*K_D_* = 34.77 ± 11.8 nM), with a vertical orientation, in comparison to parent PG, which exhibited no measurable binding affinity. Incorporation of the linker in the fusion protein, LPG, had no effect on the antibody-binding function of PG, which retained its secondary structure and displayed no alteration of its chemical stability. The LPG system provided a milder, easier, and faster affinity-driven immobilization of antibodies to inorganic surfaces when compared to traditional chemical coupling techniques.

## 1. Introduction

The immobilization of proteins to solid surfaces is a key factor for many biological applications, including the development of biosensors and biocompatible materials. It is primarily achieved through a firm linkage between the functional protein and the solid surface. Protein immobilization onto solid supports is a very laborious task owing to the heterogeneous nature of proteins and their structural dynamics. A successful immobilization should involve mild physical and chemical conditions for the binding of biomolecules with very little or no non-specific binding in order to maintain the protein’s biological functionality. Traditionally, the most widely used immobilization method is covalent attachment of proteins to the reactive groups of solid matrices via the protein’s primary amines and carboxylic acids [1,2]. One of the major drawbacks of this coupling method is the potential attachment of biomolecules in a random orientation, which may limit or cause complete loss of the protein’s biological function [3].

Solid-binding peptides (SBPs) are short amino acid sequences that display binding affinity towards the surface of a variety of materials such as metals, semiconductors, carbon materials, polymers, and minerals [4,5,6,7]. Unlike conventional bioconjugation methods (e.g., covalent attachment), SBPs can act as molecular binders to direct the immobilization and orientation of proteins onto solid supports without impeding their functionality.

A number of factors play an important role in determining the exact binding mechanism of SBPs onto a solid surface. These include (i) the charge, composition, sequence, and structural conformation of the SBP; (ii) the physical and chemical properties of the solid surface of interest such as its chemistry, charge, size, and topography; (iii) the nature and condition of the surrounding media; and (iv) molecular dynamics of the SBP–solid interface. Based on all these parameters, an SBP diffuses and reorients on a solid surface to attain its lowest energy conformation(s) [8,9].

Several SBPs have been studied as a function of the above physical and chemical properties using various biophysical characterization techniques. For example, Seker et al. [10] showed that the adsorption and structural features of SBPs can be adjusted by the presence or absence of molecular constraints. Based on the data obtained from surface plasmon resonance (SPR) and circular dichroism (CD) spectroscopy, it was found that the binding and conformation of a platinum (Pt)-binding septapeptide in its linear and cyclic forms were purely dependent on the cysteine–cysteine (C–C) residues in the peptide sequence. SPR studies performed by Matsuno et al. [4] showed for the first time that a poly (L-lactide) (PLLA)-binding heptapeptide distinguished between the 3D arrangement of functional groups in the crystal lattice of PLLA polymorphs. It was also found that the binding of the peptide to the PLLA surface was primarily due to the proton-donor amino acids histidine (H), lysine (K), arginine (R), and aspartic acid (D) that formed H-bonds with the ester groups of the PLLA backbones. The binding specificity was largely due to the hydrophobic interactions between alanine (A) and leucine (L) residues of the peptide and the methyl and methine groups of the PLLA. Sultan et al. [11] demonstrated that the binding of peptides to the solid surface was mediated by the ionic strength of the solvents, which further determined the nature and structure of the adsorbed peptide layer. Quartz crystal microbalance with dissipation monitoring (QCM-D) analysis performed on two distinct titanium (Ti)-binding peptides of different overall charge and hydrophobic residues showed that while water as the solvent leads to the formation of viscoelastic multilayers on the surface, a 0.15 M NaCl saline solution directed the formation of a rigid monolayer on the surface for both the peptides studied.

The amino acid sequence and surrounding chemical environment of any peptide determine its secondary structure. By using CD spectroscopy in combination with site-directed alanine (A) scanning, Sawada and co-workers found that the proline (P) residues present in the native state of a naphthalene-binding peptide (Nap01) gives the peptide a β-turn structure and that these residues were necessary for strong naphthalene binding [12]. We previously developed an SBP-based bioconjugation platform using a genetically engineered fusion protein, linker-protein G (LPG) [13]. LPG was designed to contain two functionally distinctive regions—a silica-binding SBP (referred to as the “linker”) and an antibody-binding protein (protein G, PG) [13]. The linker was made of a 4 × 21 amino acid sequence repeat ((VKTQATSREEPPRLPSKHRPG)_4_VKTQTAS) that displayed high binding affinity towards silica-based materials. In this platform technology, LPG acts as an anchor for the rapid and oriented immobilization of antibodies onto silica surfaces, thereby ensuring the functional display of conjugated antibodies without the need for complex conjugation chemistry. Although this linker technology has been widely used in several biotechnology and biomedical applications [14,15,16,17], there is still a lack of understanding about the interaction mechanism which facilitates the binding of this SBP to the silica surface.

In this work, different biophysical characterization techniques, namely QCM-D, SPR, CD, and fluorescence spectrometry, were used for a detailed study of the binding of LPG to silica. In addition, the effects of the linker on the stability and antibody-binding function of the parental PG were also investigated using CD and fluorescence spectrometry.

## 2. Materials and Methods

### 2.1. Materials and Chemicals

SiO_2_-coated crystals for QCM-D were purchased from ATA Scientific (Taren Point, NSW, Australia). CM5 chips were purchased from GE Healthcare (Parramatta, NSW, Australia). Recombinant PG from *Streptococcus* sp., fetal bovine serum (FBS), and human serum were purchased from Sigma-Aldrich (Castle Hill, NSW, Australia). Humanized anti-HER2 monoclonal antibody trastuzumab was obtained from Jomar Life Research (Melbourne, Australia) and the human HER2/ErbB2 protein (His-Tag) was ordered from Sino Biological Inc. (Beijing, China). All biological assays were performed at room temperature with standard phosphate-buffered saline (1 × PBS) at pH 7.4 containing 10 mM Na_2_HPO_4_, 1.8 mM KH_2_PO_4_, 137 mM NaCl, and 2.7 mM KCl. All other chemicals were purchased from Sigma-Aldrich unless otherwise stated. Murine monoclonal antibody, G203 (IgG1), was purchased from BTF Pty Ltd. (Macquarie Park, NSW, Australia). Purified LPG was obtained as described previously [13]. The 21-single peptide LP1 (VKTQATSREEPPRLPSKHRPG) was synthesized by Mimotopes (Mulgrave, VIC, Australia).

### 2.2. Circular Dichroism (CD) Spectroscopy

CD spectra were collected at room temperature (approximately 25 °C) on a JASCO J-1500 spectropolarimeter (JASCO Corporation, Tokyo, Japan). PBS absorbs strongly at wavelengths below approximately 200 nm, which precludes collection of CD data at these wavelengths. Hence, all CD data were collected in water. LPG and PG were buffer exchanged to Milli-Q water using Amicon Ultra-10K 0.5 mL centrifugal filters (Merck Millipore, Bayswater, VIC, Australia). Stock solutions of each protein were then further diluted in Milli-Q water to a final concentration of 0.1 mg/mL for CD spectroscopy. Wavelength scans were performed between 180 and 350 nm in a rectangular, 1 mm pathlength, quartz cuvette (Starna Scientific Ltd., Ilford, UK). For each sample, three accumulations were recorded using a 2 nm bandwidth, a scan speed of 100 nm/min, and a digital integration time (DIT) of 2 s. The data are reported in terms of mean residue ellipticity (θ_M_), expressed in deg·cm^2^·dmol^−1^·residue^−1^.

### 2.3. Fluorescence Spectroscopy

LPG and PG were dissolved to a final concentration of 2 µM in 1 × PBS containing various concentrations (0–7 M) of guanidinium hydrochloride (GdnHCl). Fluorescence spectra were recorded on a JASCO FP-8500 spectrofluorometer (JASCO Corporation, Tokyo, Japan). All measurements were recorded at room temperature (approximately 25 °C) in a micro-volume fluorescence cuvette with 3 mm pathlength (Starna Scientific Ltd., Ilford, UK). Fluorescence emission spectra were collected between 300 and 550 nm using a 295 nm excitation wavelength, 2.5 nm excitation bandwidth, 5 nm emission bandwidth, and scan speed of 100 nm/min.

### 2.4. Surface Plasmon Resonance (SPR)

Surface plasmon resonance (SPR) experiments were conducted on a BIAcore 2000 instrument (GE Healthcare, Uppsala, Sweden) using the most common, versatile, and commercially available sensor chips (i.e., CM5 sensor chips) covered with a carboxymethylated dextran layer. Measurements were performed in filtered and degassed HEPES-buffered saline, HBS-P buffer (10 mM HEPES pH 7.4, 150 mM NaCl, 0.005% (*v*/*v*) surfactant P20); the same running buffer was used for protein dilutions. In the first step, the surface of the CM5 chip was activated by suitable amine coupling chemistry, using 1-ethyl-3-(3-dimethyl-aminopropyl)-1-carbodiimide hydrochloride (EDC) and *N*–hydroxysulfosuccinimide (EDC/NHS) and ethanolamine to deactivate unreacted NHS-esters. A maximum response level of 2750 RU was achieved by diluting the G203 (IgG1) in 10 mM CH_3_COONa (pH 4.0) to a final concentration of 10 µg/mL. For kinetic studies, both PG and LPG were serially diluted in HBS-P buffer to a final concentration range of 1.45–23.1 nM. The system was equilibrated with running buffer until a stable baseline was obtained, after which the samples were automatically injected in duplicates for 3 min (association) at a flow rate of 20 µL/min at room temperature, followed by a 5 min dissociation. The sensor surface was regenerated by injecting glycine-HCl pH 2.0 for 30 sec at 20 µL/min. Between each sample injection, a re-equilibrium was established between the sensor surface and running buffer by a 90 s pre-run phase. A similar experiment was performed with LP1 to determine any non-specific interaction with the antibody. Global fitting of the resulting sensorgrams at five different concentrations was done using BIAevaluation software version 4.1 (GE Healthcare, Uppsala, Sweden), which simultaneously fits all sensorgrams and evaluates both the association (*k_a_*) (M^−1^s^−1^) and dissociation (*k_d_*) (s^−1^) rate constants. The binding affinity (*K_D_*) (M) was calculated by the equation *K_D_* = *k_d_*/*k_a_*. A similar procedure and experimental setup were adopted for the interaction of PG and LPG with anti-HER2 monoclonal antibody trastuzumab. Data were collected by performing each experiment in triplicate and the final values were reported as the mean of all three experiments.

### 2.5. Quartz Crystal Microbalance with Dissipation Monitoring (QCM-D)

A QSense E4 system (Biolin Scientific AB, Gothenburg, Sweden) was used to quantify the adsorption and binding strength of LPG to silica. AT-cut SiO_2_-coated QCM-D quartz crystal sensors were excited at a fundamental frequency of 5 MHz as well as 3rd, 5th, 7th, 9th, 11th, and 13th overtones, and changes in frequency (Δ*f*), and dissipation (ΔD) were recorded. Additionally, 1 × PBS buffer pH 7.4 at room temperature was used as the flow medium and to prepare the samples. Before starting the measurements, the sensors were first treated in a UV-ozone chamber (Diener Electronic GmbH, Ebhausen, Germany) for 15 min followed by immersion in a solution of 2% (*w*/*v*) SDS and then incubated in a water bath for 20 min at 60 °C. The sensors were moved to another beaker with fresh Milli-Q ultra-pure water (Merck Millipore, Bayswater, VIC, Australia) and incubated further for 20 min. The sensors were carefully rinsed with Milli-Q water, dried under N_2_ stream, and treated with UV-ozone for 15 min. The sensors were mounted in the modules provided in the Q-Sense instrument and 1 × PBS buffer was flowed (150 µL/min) over the bare sensor surface until a flat baseline was obtained. The protein solutions were made in 1 × PBS buffer and injected over the sensors through the flow cell at the same flow rate. The protein samples were made to flow until the system reached equilibrium, or until no change was observed in Δ*f*. Finally, 1 × PBS was injected again to wash the sensor surface as well as any unbound proteins. Several protein concentrations (3.27–654 nM) were prepared in 1 × PBS, and their Δ*f* was measured to determine the dissociation constant or binding affinity (*K_D_*). The values obtained were fitted using the Langmuir adsorption model. Data were collected from three independent measurements under the same conditions and parameters.

### 2.6. Adsorption Isotherms

The equilibrium binding constant (*K_D_*) and binding affinity of the proteins to silica were obtained using the GraphPad Prism 7 software (GraphPad Software, La Jolla, CA, USA). The one-site-specific binding model was used to fit the adsorption isotherm of the binding interactions between LPG and the SiO_2_-coated QCM-D sensor crystals. The one-site binding (hyperbola) was defined by the following:

Δ*m* = (*B_max_* × *C*)/(*K_d_* + *C*), (1)

where Δ*m* is the amount of adsorbed analyte, *C* is the concentration of the analyte solution, *B_max_* is the maximum adsorption of analyte onto the surface, and *K_d_* is the apparent dissociation constant.

### 2.7. Viscoelastic Properties of Adsorbed Proteins

The Δ*f* measurements obtained from the QCM-D are related to the binding of molecules to the sensor surface. However, a linear relationship between Δ*f* and mass is only valid for rigid layers. In this case, the adsorbed area mass (Δ*m*) is proportional to the Δ*f* in the case of a rigid film, where Δ*D* < 1 × 10^−6^ per 10 Hz. In such cases, the Sauerbrey equation (Δ*m* = – *C* • Δ*f_n_*/*n*) can be applied, where *C* is the constant of mass sensitivity (17.7 ng Hz^−1^ cm^−2^) for a crystal with 5 MHz fundamental frequency and *n* is the number of frequency overtones (*n* = 3 was used in this work). However, biological systems usually display viscoelastic properties that are measured by ΔD and will result in the Sauerbrey equation underestimating the adsorbed mass. Capturing Δ*f* and ΔD measurements at multiple harmonics enables modelling of the data with the Voigt viscoelastic model incorporated in QSense software’s QTools (Biolin Scientific AB, Gothenburg, Sweden) to obtain parameters including mass, thickness, density, viscosity, and storage modulus [18]. In our work, Δ*D* > 1 × 10^−6^ per 10 Hz, indicating a viscoelastic layer, hence the Voigt model was used [19,20,21] to calculate the viscoelastic properties as well as the adsorbed mass of the peptide on the sensor surface.

### 2.8. Chemical Biofunctionalization

Prior to silanization, the QCM-D sensor crystals were cleaned and treated with oxygen plasma at a maximum power for 3 min in a Pico Plasma system (Diener Electronic GmbH, Ebhausen, Germany). The silanization solution was prepared by the addition of 3-aminopropyl-triethoxysilane (APTES) to 95% ethanol to obtain a solution concentration of 5% APTES by volume. Silanization was performed at room temperature for 10 min. After silanization, probes were flushed with 95% ethanol, ultrasonicated for 3 min in 95% ethanol, followed by final rinsing in Milli-Q water. APTES-modified surfaces were activated with 1% glutaraldehyde (GA) in 1 × PBS for 60 min before immobilization. The modified QCM-D sensor crystals were placed in the Qsense E4 analyzer and 1 × PBS was injected at a flow rate of 150 μL/min until a stable baseline was achieved. PG at a final concentration of 0.654 μM in 1 × PBS was injected at the same flow rate until the Δ*f* was stable (reached saturation), after which the crystal was washed again with 1 × PBS to remove unbound proteins. Any remaining active groups were deactivated after incubation for 15 min with a solution containing 0.1 M Tris and 50 mM ethanolamine (pH 9.0) followed by a blocking step (1 mg/mL bovine serum albumin, BSA, 10 min) to reduce non-specific binding. Humanized anti-HER2 monoclonal antibody trastuzumab was prepared at a final concentration of 1 μg/mL in 1 × PBS and injected at a similar flow rate to achieve a stable response. Finally, the HER2 antigen at a concentration of 1 μg/mL was injected until a stable Δ*f* was obtained. Between each step, the crystals were thoroughly rinsed with 1 × PBS.

## 3. Results and Discussion

### 3.1. Effect of the Linker on the Structure and Stability of Protein G

**Figure 1:**
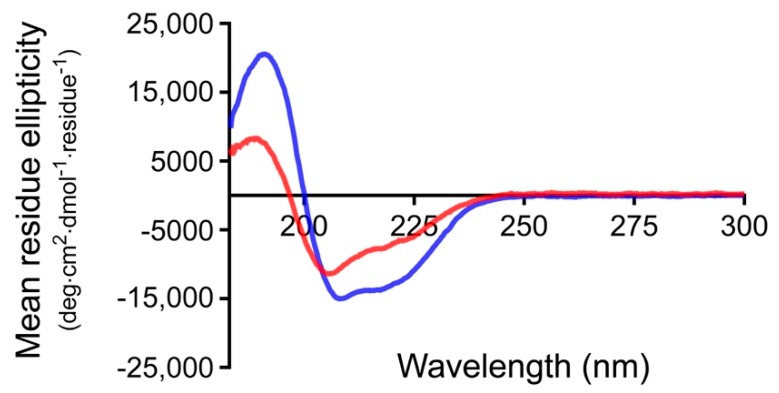
Far-UV spectra of *Streptococcus* protein G (PG) (blue) and linker-protein G (LPG) (red) in water at a concentration of 0.1 mg/mL.

Circular dichroism (CD) was used to determine if the presence of the linker affects the secondary structure of PG. The far-UV CD spectra of PG and LPG were measured at a concentration of 0.1 mg/mL (small variations in concentration were accounted for by scaling the CD signal based on the absorbance measured between 180 nm and 350 nm). As can be seen in Figure 1, both proteins display a positive peak at ~190 nm and negative peaks at ~208 and 222 nm, consistent with a significant α-helical folded structure. Furthermore, CD secondary structure fitting of this data was performed using Dichroweb [22], using the SELCON3 algorithm [23] and the reference Set 3 [24,25] (which is optimized for a wavelength range of 185–240 nm). The proportion of α-helix is estimated to be ~36% for PG and ~22% for LPG, respectively. As the PG module (185 amino acids) accounts for only ~2/3 of the total LPG protein (276 amino acids), assuming the linker itself does not adopt any additional helical structure, this result is consistent with the PG module in PG and LPG maintaining the same fold. These results are also similar to those obtained by Goward et al. whose calculated secondary structure gives ~29% α-helix for PG [26]. The LPG linker has been previously predicted by homology modeling to be unfolded [16]. Interestingly, using the SELCON3 algorithm and reference Set 3 (as well as many other combinations of fitting algorithm and reference set available on Dichroweb), the proportion of β-sheet was estimated to be ~17% for PG and ~27% for LPG, respectively. Taken at face value, this result would suggest that the linker assumes β-sheet structure in the presence of PG. However, it should be noted that random coil and β_II_ structure are, unfortunately, indistinguishable by CD. The reference sets used for CD secondary structure deconvolution typically consist of globular proteins where β_II_ structures are often generically assigned as β-sheet. Hence, CD secondary structure fitting methods can have trouble distinguishing between unstructured regions and β_II_ structures, particularly in proteins that contain large disordered regions [27]. While it is tempting to suggest that the greater proportion of β-sheet estimated for LPG in comparison to PG may be consistent with the linker lying along the surface of the PG module in a β-sheet conformation, we cannot unequivocally conclude whether the linker truly adopts β-sheet structure in the presence of PG or remains unstructured.

**Figure 2:**
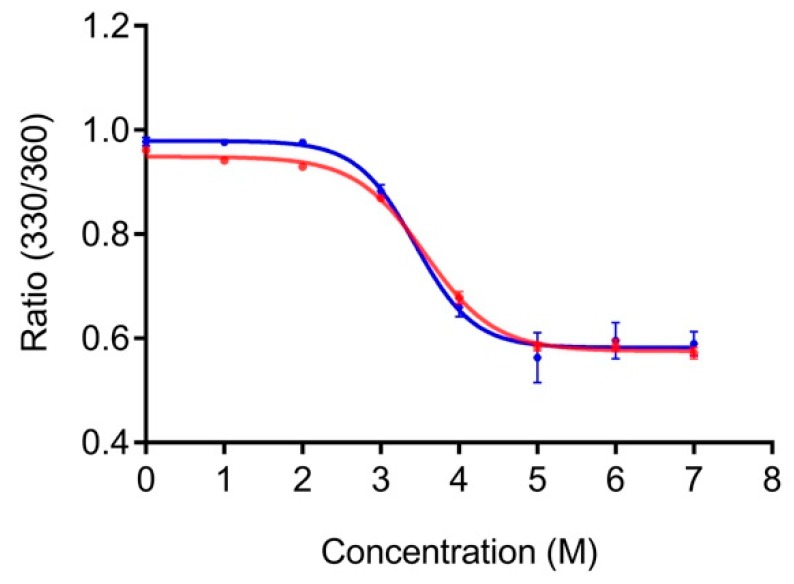
Unfolding of PG (blue) and LPG (red) in guanidinium hydrochloride (GdnHCl). Both PG and LPG were diluted to a final concentration of 2 µM in 1 × PBS, pH 7.4, containing various concentrations of GdnHCl.

PG is stable at high temperatures (melting temperature >80 °C) [26]. Hence, performing thermal unfolding studies to compare the stability of PG and LPG would, in practice, be challenging if not impossible. Instead, we used fluorescence spectroscopy to compare the relative stability of PG and LPG to chemical denaturant, namely, GdnHCl. PG contains eight tyrosine and three tryptophan residues, whereas the linker itself lacks any of these aromatic amino acids. Our fluorescence experiments were conducted using an excitation of 295 nm to minimize excitation of the tyrosine residues, which generally occurs between 280 and 290 nm [26]. The linker sequence does not have any aromatic residues, so the overall fluorescence of LPG and PG with and without the linker can be attributed to the tryptophan residues of PG. Tryptophan fluorescence is environmentally sensitive, with the wavelength of maximum fluorescence emission correlating to the folding state of the protein, that is, hydrophilic environment fluorescence occurs at longer wavelengths. Unfolding of LPG and PG without the linker was monitored by the shift in maximum tryptophan fluorescence emission to longer wavelength with increasing GdnHCl concentration. The ratio of fluorescence emission intensity at 330 nm and 360 nm (330/360) was plotted against GdnHCl concentration and the data were non-linearly fitted, assuming a two-state denaturation, using GraphPad Prism 7.0 software (Figure 2).

The GdnHCl denaturation curves for PG and LPG are shown in Figure 2. The relative fluorescence intensities at 330 nm and 360 nm (330/360) was used to monitor the shift in fluorescence emission maxima after excitation at 295 nm. No change in tryptophan fluorescence is seen until approximately 2 M GdnHCl for both LPG and PG. Similarly, both proteins were maximally unfolded after approximately 5 M GdnHCl. Based on the non-linear, two-state fitting of the denaturation curves, the concentration of GdnHCl required to induce 50% unfolding was determined to be 3.4 ± 0.2 M (95% CI) for PG and 3.6 ± 0.1 M (95% CI) for LPG. These results indicate that both PG and LPG have similar chemical stabilities, and thus, the linker region of LPG does not significantly affect the structural stability of PG.

### 3.2. Effect of the Linker on the Antibody-Binding Function of Protein G

**Figure 3:**
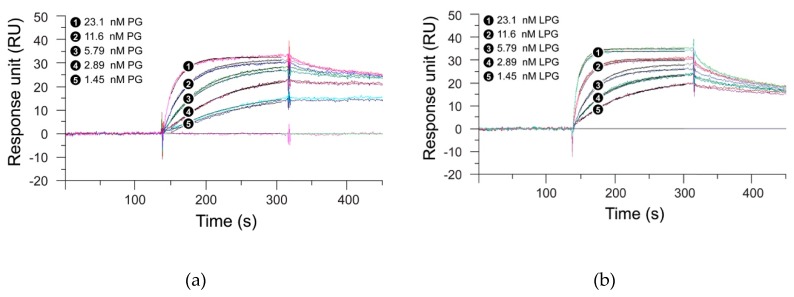
Global fit of 1:1 Langmuir binding model with trastuzumab for (**a**) PG and (**b**) LPG. Black lines constitute two repeat injections of PG and LPG over trastuzumab immobilized on a surface. The concentrations of each analyte (PG and LPG) injected were 23.1, 11.6, 5.79, 2.89, and 1.45 nM. Experiments were performed over a 700 s period, but for graphical display only the first 450 s are shown. Dissociation was performed for a total of 5 min.

**Figure 4:**
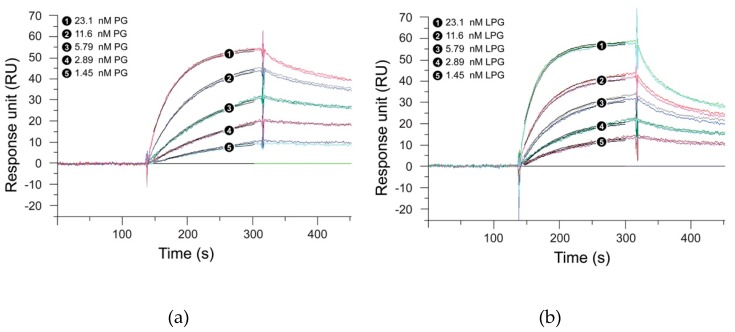
Global fit of 1:1 Langmuir binding model with G203 (IgG1) for (**a**) PG and (**b**) LPG. Black lines constitute two repeat injections of PG and LPG over G203 (IgG1) immobilized on a surface. The concentrations of each analyte (PG and LPG) injected were 23.1, 11.6, 5.79, 2.89, and 1.45 nM. Experiments were performed over a 700 s period, but for graphical display only the first 450 s are shown. Dissociation was performed for a total of 5 min.

SPR measurements were performed to analyze whether the linker had any effect on the antibody-binding function of the *Streptococcus* PG. For this purpose, the binding kinetics of PG with the linker (LPG) and PG without the linker were studied with two antibodies—trastuzumab and G203 (IgG1).Trastuzumab, a humanized IgG1 monoclonal antibody, targets the HER2 receptor that plays an important role in normal cell growth and differentiation, whereas G203 is a murine monoclonal antibody, also IgG1, which targets *Giardia lamblia*, a microscopic parasite, present on the cyst walls of the small intestine. The basic purpose of using these two different antibodies was to study whether the two proteins under study show similar binding interactions, irrespective of the source and target of the antibody. Figure 3; Figure 4 represent the SPR sensorgrams for the specific binding interactions of PG and LPG to trastuzumab and G203 (IgG1) using a 1:1 Langmuir approach. The Langmuir approach is based on the fact that a monolayer has been formed on a homogeneous surface, and thus, equivalent binding sites are available, meaning one antibody binds to one protein and that it is independent of the interactions from the occupancy of the nearby binding sites [28]. Since our SPR data fit the 1:1 Langmuir model, we assumed a monolayer adsorption of proteins to antibodies and, hence, a well-defined covering. The chi-squared (χ^2^) value (a measure of fitting reliability) was less than 5 for each of the kinetic fitted results. According to the BIAevaluation handbook, a χ^2^ value of less than 10 represents an excellent-fit model [6].

Table 1 presents the association and dissociation rates derived from the SPR measurements for the binding of PG and LPG to trastuzumab and G203 (IgG1), respectively, using a 1:1 Langmuir binding model. Figure 3 and Figure 4 indicate that PG had a faster association but a slower dissociation for both the antibodies when compared to LPG, which displayed fast association with an equally fast dissociation. However, quantitative data from Table 1 suggest that both proteins (PG and LPG) associate and dissociate at similar rates (i.e., all the *k_a_* and *k_d_* have a magnitude of 10^6^ M^−1^s^−1^ and 10^–3^ s^−1^, respectively). The different trends for the qualitative and quantitative data can be attributed to random orientation of the antibodies conjugated to the gold surface of the SPR chip. In addition, PG and LPG displayed strong and similar binding affinities to both the antibodies in the nM range (*K_D_* = 10^–9^ M).

The lack of measurable SPR signal response for the linker alone (Figure S1, Supplementary Materials) suggests that non-specific binding interactions do not occur between the linker and the antibody. From the SPR experiments, we conclude that the linker has minimal influence on the overall binding function of PG and that only the PG part of LPG is responsible for antibody binding. Moreover, the presence of the linker does not appear to significantly affect the stability of the PG–antibody complex as indicated by the similar half-lives measured for both proteins, LPG and PG (Table 1). The half-life values also suggest that the proteins will not diffuse too far and will not be involved in an unintended process that will reduce the crosstalk between the adjacent protein molecules.

### 3.3. Binding Kinetics of LPG

**Figure 5:**
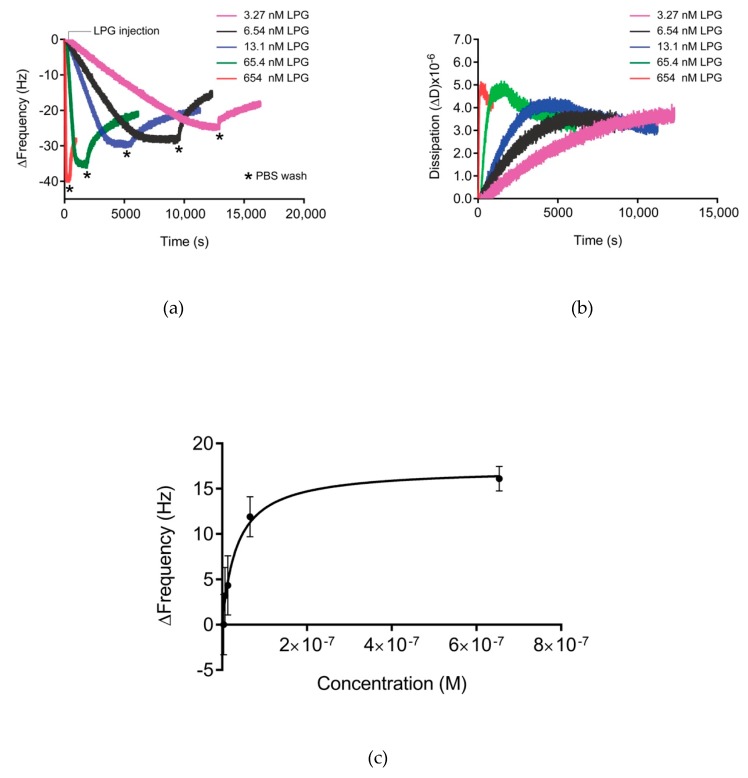
(**a**) Δ*f* and (**b**) ΔD for the 3rd overtone of the absorbed LPG on silica-coated QCM-D crystals at various concentrations (3.27–654 nM). The measurement consists of three steps with flow rates of 150 µL/mL—baseline formation (PBS buffer), adsorption (LPG in PBS buffer, flow until saturation is was achieved), and washing (PBS buffer) to remove unbound LPG. Three independent measurements were performed for each concentration. (**c**) Langmuir adsorption isotherm for the adsorption of LPG to silica. The data were fitted using the single-site-specific binding model.

The binding affinity of LPG to silica was calculated using QCM-D measurements (Figure 5a,b). The Langmuir fitted data (Figure 5c) gave an apparent dissociation constant/binding affinity (*K_D_*) of 34.77 ± 11.8 nM, which is comparable to the binding affinity reported for other silica-binding peptides [29,30,31]. When PG was used at the same molar concentrations as LPG, no measurable Δ*f* was observed in the QCM-D sensorgram (Figure S2, Supplementary Materials). Accordingly, no measurable binding affinity was calculated. Initial qualitative binding assays using SDS-PAGE implied that PG without the linker sequences was unable to bind to silica [13].

**Figure 6:**
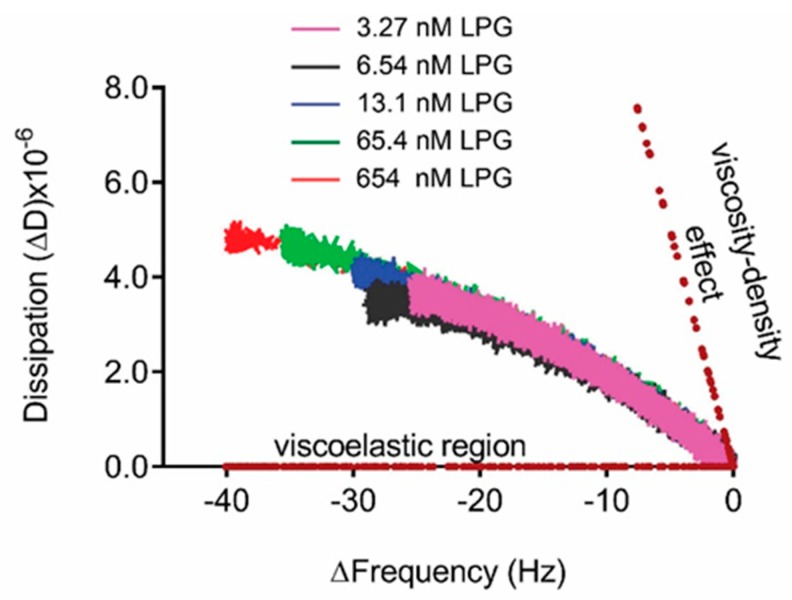
D*f* plot (3rd overtone) for the binding interactions between LPG and silica surface. The dotted lines indicate the pure elastic mass and viscosity–density responses.

Presentation of the QCM-D data in Figure 5 as ΔD versus Δ*f* (*Df* plots) enabled the comparison of adsorption behavior for different concentrations of LPG (Figure 6). D*f* plots depend dynamically on the effective deposited mass, its viscoelastic properties, the structure of the protein, and how these features evolve over time during interactions. The region between the dotted lines in the D*f* plot represents the viscoelastic region between a pure elastic mass response where ΔD = 0 and a pure liquid viscosity–density change in the fluid above the crystal in the absence of surface binding [19,32,33]. The binding interactions between different LPG concentrations and silica surface resulted in a D*f* plot that fits within the viscoelastic region. Over the concentrations of LPG tested, the dynamic adsorption behavior was the same as shown by the similarly shaped profile for each concentration, with higher concentrations resulting in higher deposited mass, as shown by the larger Δ*f* values at the end of the adsorption phase. These data demonstrate the stability of the LPG binding over the concentrations tested. Other globular proteins display similar stability of binding to surfaces over a range of concentrations, such as albumin binding to platinum [34]. However, it should be noted that the shape and magnitude of the Δ*f* and ΔD values are different due to different protein properties such as size and isoelectric point that govern the kinetics of interfacial interactions.

The ΔD measurements obtained (Figure 5b and Figure 6) were greater than 1 × 10^−6^ per 10 Hz, indicating that the adsorbed layer was viscoelastic necessitating the use of the Kelvin–Voigt viscoelastic model to estimate adsorbed mass, thickness, and layer viscosity (Table 2). This model is based on the hypothesis that the adsorbed layer is of uniform density and thickness, does not flow, and maintains its shape. The raw data for the LPG interaction with silica displayed variation in the ΔD values between different overtones, with the third overtone (*n* = 3) displaying higher ΔD values in comparison to the fifth (*n* = 5), seventh (*n* = 7), and eleventh (*n* = 11) overtones (Figure S3, Supplementary Materials). Table 2 presents the various viscoelastic parameters for the binding interactions between LPG and the silica-coated QCM-D crystal at the association and dissociation phases obtained from modelling the data by applying the Kelvin–Voigt model using the 3rd overtone.

The maximum thickness of the peptide layer calculated by the Kelvin–Voigt model was 7 nm, and the raw data for both the thickness and viscosity (Figure S4, Supplementary Materials) correlate with each other. This observation further supports the formation of a viscous multilayer of peptides on the surface of the silica. The viscous nature of the peptide layers can also be explained by the system response observed upon introduction of the washing buffer (PBS). The soft and viscous layer formed was reduced in size upon rinsing, suggesting the removal of a loosely bound peptide. This can be explained by the fact that the silica-binding peptide sequence used for our studies has a considerable number of hydrophobic amino acid residues (26 residues out of a total of 91 residues). The PBS washing step reduced the Δ*f* and ΔD consistent with the removal of loosely bound peptides via disruptions of the hydrophobic bonds to form a stable multilayer structure. In a similar study, a purely hydrophobic silica-binding peptide was reported to form a multilayer on silica via hydrophobic binding [35]. The authors found a multilayer formation on silica for a purely hydrophobic silica-binding peptide and concluded that it was not only the nature of the peptide itself, but also the hydrophobicity/hydrophilicity of the surface which governed the binding interaction between the two [35]. A monolayer of LPG (30.4 kDa) was approximated to 350 ng/cm^2^ for an end-on orientation and 190 ng/cm^2^ for a side-on orientation on a dry mass basis when assuming LPG to be a globular protein approximately half the size of albumin (66 kDa) due to its molecular weight being approximately half. The 7 nm thick layer of immobilized LPG equates to approximately 770 ng/cm^2^ that was hydrated and viscoelastic in nature. Adsorbed mass estimates from QCM-D include the bound water associated with the proteins and, for globular proteins, the hydration is reported to account for approximately 50% of the mass [36]. This together with the relatively low dissipation values observed during LPG adsorption to silica suggested that LPG adsorbed to silica predominantly in an end-on orientation. The end-on-end peptide orientation is an advantage for immobilizing proteins on the surface since the PG part of the LPG is free and oriented towards the solution phase, enabling maximal interaction with the antibody and, thus, reducing the chances of non-specific binding. The maximum amount of LPG bound to the surface of the silica-coated QCM-D crystal was determined to be 863 ng/cm^2^ for 20 µg/mL LPG as this is the maximum concentration of LPG sufficient to saturate the entire surface of the silica crystal. Considering the molecular mass of LPG (30.4 kDa) and the mass of one LPG molecule (5.05 × 10^−11^ ng), there are 1.7 × 10^13^ LPG molecules bound per cm^2^ of silica.

**Figure 7:**
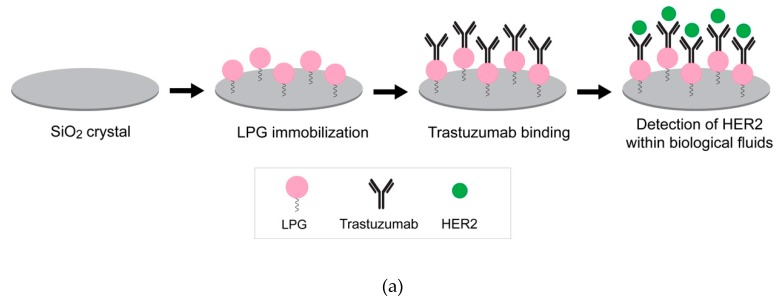

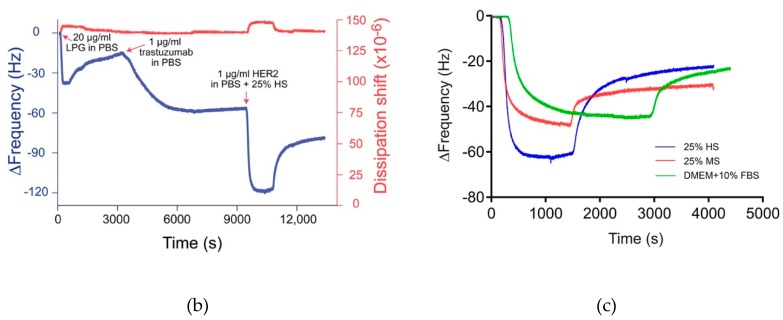
(**a**) Experimental workflow to determine the interaction of antibody (trastuzumab) to silica-immobilized LPG. (**b**) QCM-D response for the binding interactions of trastuzumab to silica-immobilized LPG and subsequent detection of HER2 antigen spiked in 25% human serum. The arrows indicate the time points for the injection of LPG, trastuzumab, and HER2. (**c**) Frequency shifts for binding interactions between trastuzumab and HER2 spiked in various biological fluids. Extensive PBS washing was performed between each sample injection.

An orientation on the surface which makes the binding site available is essential to preserve the bioactivity of immobilized biomolecules. The QCM-D results indicated that LPG binds strongly to silica and forms viscoelastic multilayers with an end-to-end conformation. Further studies were performed to support this finding using an antibody–antigen binding assay. The interaction between silica-immobilized LPG and trastuzumab was determined in various biological fluids (i.e., human serum, mouse serum, and FBS) spiked with the HER2 antigen (Figure 7a). These experiments also provided information about the binding efficiency of the system in the presence of common and more complex biological environments used for clinical biosensing.

The binding interactions between the immobilized LPG and trastuzumab are shown in Figure 7b. The binding of trastuzumab to LPG is represented by the decrease in Δ*f*. The binding interaction between both molecules was strong since only a negligible Δ*f* was observed after the unbound antibody was washed off. HER2 can bind to the immobilized trastuzumab in buffer which acts as a positive control. Relative binding to this positive control can then be established when HER2 is presented in more complex mixtures including serum. The interaction between trastuzumab and HER2 spiked in 25% human serum and mouse serum as well as DMEM + 10% FBS is shown in Figure 7b and Figure S5, Supplementary Materials. In both the figures, a strong drop in the Δ*f* was observed when HER2 spiked in different serums was injected in the QCM-D, indicating a strong binding of HER2 to trastuzumab. Figure 7c represents the Δ*f* for the binding interactions between trastuzumab and HER2 spiked in different biological fluids. HER2 binds to trastuzumab irrespective of the buffer, with the highest Δ*f* observed in human serum, followed by mouse serum and FBS. The minimum binding interaction in the case of FBS is probably due to the unstable composition of the serum media which may interfere in the efficient detection of HER2. Moreover, there may be some non-specific interactions due to the presence of foreign proteins (e.g., antigens) in FBS. The advantage of human serum over FBS has also been demonstrated in cell culture studies by Heger et al. [37], where human serum remarkably enhanced the invasion and spheroid formation when compared to FBS. In conclusion, with respect to our studies and irrespective of the buffer media, the responses indicate that the HER2 was bound to LPG in such an orientation that its antigen-binding fragments (Fab) were exposed to the surrounding environment.

### 3.4. Linker and Covalent Functionalization of Silica Surface

The efficiency of the LPG-mediated functionalization was compared to the traditional amine coupling method used to covalently bind PG onto the silica-coated QCM-D crystal. Covalent attachment of PG was required for these studies as PG alone did not bind to silica (Figure S2). A glutaraldehyde (GA) linker was used to chemically immobilize PG to an aminated silica surface. Prior to PG conjugation, an amino reactive aldehyde layer was formed on the silica-coated QCM-D crystal by first treating it with oxygen plasma followed by reaction with 3-aminopropyltriethoxysilane (APTES), and finally derivatizing with GA. The length and conditions of the reaction can be altered to control the thickness of the silane layer. After the chemical coupling of PG, a small amount of ethanolamine (in Tris buffer) was added to deactivate the unreacted aldehyde groups, followed by the addition of BSA to block non-specific binding sites. Each of the above-mentioned steps included a thorough washing step with Milli-Q water. The same experimental procedure was used to calculate the viscoelastic parameters for the binding of antibodies to PG. The QCM-D sensorgrams for the Δ*f* and ΔD for three independent measurements are shown in Figure S6, Supplementary Materials, and they revealed that the chemical immobilization of PG resulted in a smaller change in both *f* and D. Modelling these measurements in the Kelvin–Voigt model indicated that chemically immobilized PG formed a thinner layer than physisorbed LPG (Table 3). In addition, the chemically immobilized PG layer supported approximately 38% of the amount of antibody that the LPG layer supported (Table 3), which in turn bound approximately 36% of the amount of antigen as the LPG layer. These data suggest that LPG immobilization on silica was a more efficient method of antibody and antigen capture than chemically immobilized PG. The linker-mediated immobilization was experimentally easy and only took ~6 min. On the contrary, the chemical conjugation had longer experimental time (~3 h) and involved the handling of harsh chemicals.

## 4. Conclusions

The binding interaction experiments conducted with LPG and PG on silica provided new quantitative data that support previous observations reported by Sunna et al. [13] based on qualitative data. The binding of the LPG to the silica surface is purely mediated by the silica-specific linker region and was determined to be in the nM range. The results from QCM-D suggest that the linker orientates on the surface to present PG to the soluble phase, thus enabling antibody binding. Furthermore, results obtained from various biophysical characterization techniques showed that the linker has no effect on the overall structure, chemical stability, and hence antibody-binding function of PG. Moreover, when compared to traditional chemical conjugation of biomolecules, the linker-mediated system represents a facile and rapid technology to immobilize protein on a solid surface without compromising its biological function.

## Figures and Tables

**Table 1:** Kinetic rate constants (*k_a_* and *k_d_*) and equilibrium dissociation constants (*K_D_*) for the binding interactions of PG and LPG with trastuzumab and G203 (IgG1). Data were fitted with the BIAevaluation software 4.1 using a 1:1 Langmuir binding model for three replicates of sample injection.

Protein-Antibody Complex	Rate of Association *k_a_* (M^−1^s^−1^)	Rate of Dissociation *k_d_* (s^−1^)	Equilibrium Dissociation Constant *K_D_* (M)	Half-Life t_1/2_ (s)
PG + trastuzumab	1.79 ± 0.10 × 10^6^	8.24 ± 0.92 × 10^−3^	4.62 ± 0.79 × 10^−9^	83.7
LPG + trastuzumab	3.88 ± 1.20 × 10^6^	7.48 ± 0.38 × 10^−3^	2.07 ± 0.71 × 10^−9^	92.2
PG + G203 (IgG1)	0.84 ± 0.02 × 10^6^	5.32 ± 0.29 × 10^−3^	6.30 ± 0.32 × 10^−9^	129.7
LPG + G203 (IgG1)	1.23 ± 0.06 × 10^6^	9.50 ± 0.30 × 10^−3^	7.77 ± 0.58 × 10^−9^	72.6

**Table 2:** Viscoelastic parameters for LPG and silica-binding interactions obtained by fitting the raw QCM-D data using the Kelvin–Voigt model for three sample replicates.

	Thickness (@*R_eq_*)^a^ nm	Thickness (@*k_d_*)^b^ nm	Mass Deposited (@*R_eq_*) ng/cm^2^	Mass Deposited (@*k_d_* ) ng/cm^2^	Viscosity (@*R_eq_*) × 10^−4^ kg/ms	Viscosity (@*k_d_*) × 10^−4^ kg/ms
LPG/SiO_2_	7.84 ± 0.22	5.59 ± 0.22	862.95 ± 42.43	615.13 ± 58.06	24.70 ± 0.50	20.57 ± 0.45

^a^ @*R_eq_*, frequency shift at equilibrium; ^b^ @*k_d_*, equilibrium achieved after dissociation.

**Table 3:** Comparison of the viscoelastic parameters for PG immobilization mediated by linker and through chemical conjugation and their subsequent effect on antibody and antigen binding. Each parameter is an average of three independent measurements.

Immobilization	Thickness of Bound Protein (nm)	Mass of Protein Deposited (ng/cm^2^)	Thickness of Bound Antibody (nm)	Mass Deposited for Bound Antibody (ng/cm^2^)	Thickness of Bound Antigen (nm)	Mass Deposited for Bound Antigen (ng/cm^2^)
LPG _physisorbed_	7.84 ± 0.22	862.95	20 ± 0.05	1200.14	27.84 ± 0.74	1600.47
PG _chemically immobilized_	2.89 ± 0.18	256.76	7.43 ± 0.15	450.79	12.76 ± 0.22	578.43

## Supplementary Materials

The following are available online at https://www.mdpi.com/2218-273X/10/1/4/s1, Figure S1: Global fit of 1:1 Langmuir binding model with trastuzumab for a 21-single peptide, LP1, Figure S2: Frequency shift for the absorbed PG on silica coated QCM-D crystal for various concentrations (3.27–654 nM) performed on QCM-D, Figure S3: QCM-D signal from different overtones for LPG binding to silica coated quartz crystal, Figure S4: Overlay of the Kelvin-Voigt fitted raw data for thickness and viscosity for the adsorption of LPG to silica coated crystal, *n* = 3 overtone, Figure S5: QCM-D response for the binding interactions of trastuzumab to LPG and subsequent detection of HER2 and Figure S6: CM-D response for the binding interactions of trastuzumab to PG and subsequent detection of HER2 spiked in PBS.

## References

[1] Liu G.Y., Amro N.A. (2002). Positioning protein molecules on surfaces: A nanoengineering approach to supramolecular chemistry. Proc. Natl. Acad. Sci. USA.

[2] Chan W.C.W., Nie S. (1998). Quantum dot bioconjugates for ultrasensitive nonisotopic detection. Science.

[3] Phizicky E., Bastiaens P.I., Zhu H., Snyder M., Fields S. (2003). Protein analysis on a proteomic scale. Nature.

[4] Matsuno H., Sekine J., Yajima H., Serizawa T. (2008). Biological selection of peptides for poly (l-lactide) substrates. Langmuir.

[5] Hnilova M., Oren E.E., Seker U.O.S., Wilson B.R., Collino S., Evans J.S. (2008). Effect of molecular conformations on the adsorption behavior of gold-binding peptides. Langmuir.

[6] Serizawa T., Sawada T., Matsuno H. (2007). Highly specific affinities of short peptides against synthetic polymers. Langmuir.

[7] Bansal R., Care A., Lord M.S., Walsh T.R., Sunna A. (2019). Experimental and theoretical tools to elucidate the binding mechanisms of solid-binding peptides. N. Biotechnol..

[8] Naik R.R., Stringer S.J., Agarwal G., Jones S.E., Stone M.O. (2002). Biomimetic synthesis and patterning of silver nanoparticles. Nat. Mater..

[9] Nel A.E., Madler L., Velegol D., Xia T., Hoek E.M.V., Somasundaran P. (2009). Understanding biophysicochemical interactions at the nano-bio interface. Nat. Mater..

[10] Seker U.O.S., Wilson B., Dincer S., Kim I.W., Oren E.E., Evans J.S., Tamerler C., Sarikaya M. (2007). Adsorption behaviour of linear and cyclic genetically engineered platinum binding peptides. Langmuir.

[11] Sultan A.M., Westcott Z.C., Hughes Z.E., Palafox-Hernandez J.P., Giesa T., Puddu V. (2016). Aqueous peptide–TiO2 interfaces: Isoenergetic binding via either entropically or enthalpically driven mechanisms. ACS Appl. Mater. Interface.

[12] Sawada T., Okeya Y., Hashizume M., Serizawa T. (2013). Screening of peptides recognizing simple polycyclic aromatic hydrocarbons. Chem. Commun. (Camb. U.K.).

[13] Sunna A., Chi F., Bergquist P.L. (2013). A linker peptide with high affinity towards silica-containing materials. N. Biotechnol..

[14] Liang L., Care A., Zhang R., Lu Y., Packer N.H., Sunna A., Qian Y., Zvyagin A.V. (2016). Facile assembly of functional upconversion nanoparticles for targeted cancer imaging and photodynamic therapy. ACS Appl. Mater. Interfaces.

[15] Care A., Petroll K., Gibson E.S.Y., Bergquist P.L., Sunna A. (2017). Solid-binding peptides for immobilisation of thermostable enzymes to hydrolyse biomass polysaccharides. Biotechnol. Biofuels.

[16] Sayyadi N., Care A., Connally R.E., Try A.C., Bergquist P.L., Sunna A. (2016). A novel universal detection agent for time-gated luminescence bioimaging. Sci. Rep..

[17] Shipunova V.O., Zelepukin I.V., Stremovskiy O.A., Nikitin M.P., Care A., Sunna A., Zvyagin A.V., Deyev S.M. (2018). Versatile platform for nanoparticle surface bioengineering based on SiO_2_-binding peptide and proteinaceous Barnase*Barstar interface. ACS Appl. Mater. Interfaces.

[18] Voinova M.B., Jonson M., Kasemo B. (2002). ‘Missing mass’ effect in biosensor’s QCM applications. Biosens. Bioelectron..

[19] Lord M.S., Whitelock J.M., Simmons A., Williams R.L., Milthorpe B.K. (2014). Fibrinogen adsorption and platelet adhesion to silica surfaces with stochastic nanotopography. Biointerphases.

[20] Liu S.X., Kim J.T. (2009). Application of Kevin-Voigt model in quantifying whey protein adsorption on Polyethersulfone using QCM-D. JALA.

[21] Hovgaard M.B., Dong M., Otzen D.E., Besenbacher F. (2007). Quartz Crystal Microbalance studies of multilayer glucagon fibrillation at the solid-liquid interface. Biophys. J..

[22] Whitmore L., Wallace B.A. (2004). DICHROWEB: An online server for protein secondary structure analyses from circular dichroism spectroscopic data. Nucleic Acids Res..

[23] Sreerema N., Venyaminov S.Y., Woody R.W. (1999). Estimation of the number of helical and strand segments in proteins using CD spectroscopy. Protein Sci..

[24] Sreerama N., Woody R.W. (2000). Estimation of protein secondary structure from CD spectra: Comparison of CONTIN, SELCON and CDSSTR methods with an expanded reference set. Anal. Biochem..

[25] Sreerama N., Venyaminov S.Y., Woody R.W. (2000). Estimation of protein secondary structure from CD spectra: Inclusion of denatured proteins with native protein in the analysis. Anal. Biochem..

[26] Goward C.R., Irons L.I., Murphy J.P., Atkinson T. (1991). The secondary structure of Protein G’, a robust molecule. Biochem. J..

[27] Khrapunov S. (2009). Circular dichroism spectroscopy has intrinsic limitations for protein secondary structure analysis. Anal. Biochem..

[28] Atkins P.W. (2001). Physikalische Chemie.

[29] Seker U.O.S., Wilson B., Sahin D., Tamerler C., Sarikaya M. (2009). Quantitative affinity of genetically engineered repeating polypeptides to inorganic surfaces. Biomacromolecules.

[30] Hnilova M., So C.R., Oren E.E., Wilson B.R., Kacar T., Tamerler C., Sarikaya M. (2012). Peptide-directed co-assembly of nanoprobes on multimaterial patterned solid surfaces. Soft Matter.

[31] Seker U.O.S., Sharma V.K., Akhavan S., Demir H.V. (2014). Engineered peptides for nanohybrid assemblies. Langmuir.

[32] Marx K.A. (2003). Quartz crystal microbalance: A useful tool for studying thin polymer films and comolex biomolecular systems at the solution-surface interface. Biomacromolecules.

[33] Zhou T., Marx K.A., Warren M., Schulze H., Braunhut S.J. (2000). The quartz crystal microbalance as a continuous monitoring tool for the study of endothelial cell surface attachment and growth. Biotechnol. Prog..

[34] Palmer J.C., Green R.A., Boscher F., Poole-Warren L.A., Carter P.M., Enke Y.L., Lovell N.H., Lord M.S. (2019). Development and performance of a biomimetic artificial perilymph for in vitro testing of medical devices. J. Neural Eng..

[35] Puddu V., Perry C.C. (2012). Peptide adsorption on silica nanoparticles: Evidence of hydrophobic interactions. ACS Nano.

[36] Höök F., Vörös J., Rodahl M., Kurrat R., Böni P., Ramsden J.J., Textor M., Spencer N.D., Tengvall P., Gold J. (2002). A comparative study of protein adsorption on titanium oxide surfaces using in situ ellipsometry, optical waveguide lightmode spectroscopy, and quartz crystal microbalance/dissipation. Colloids Surf. B Biointerfaces.

[37] Heger J.I., Froehlich K., Pastuschek J., Schmidt A., Baer C., Mrowka R., Backsh C., Schleußner E., Markert U.R., Schmidt A. (2018). Human serum alters cell culture behavior and improves spheroid formation in comparison to fetal bovine serum. Exp. Cell Res..

