# Supplementary material for: Elucidating the Binding Mechanism of a Novel Silica-Binding Peptide
Source: Biomolecules. 2019 Dec 18;10(1):4. doi: 10.3390/biom10010004 (PMC7022404; doi:10.3390/biom10010004)
Supplement: Supplementary file 1 [file biomolecules-10-00004-s001.pdf]

biomolecules

Supplementary Material

Elucidating the binding mechanism of a novel silica-binding peptide

Rachit Bansal [1,2], Zehra Elgundi [3], Andrew Care [1,2], Sophia C. Goodchild [1], Megan S. Lord [3], Alison Rodger [1], and Anwar Sunna [1,2,4] *

[1] Department of Molecular Sciences, Macquarie University, Sydney, NSW 2109, Australia ; rachit.bansal@hdr.mq.edu.au (R.B) ; andrew.care@mq.edu.au (A.C); sophia.goodchild@mq.edu.au (S.G); alison.rodger@mq.edu.au (A.R); anwar.sunna@mq.edu.au (A.S)

[2] ARC Centre of Excellence for Nanoscale Biophotonics, Macquarie University, Sydney, NSW 2109, Australia

[3] Graduate School of Biomedical Engineering, University of New South Wales, Sydney, NSW 2052, Australia ; z.elgundi@unsw.edu.au (Z.E) ; m.lord@unsw.edu.au (M.L)

[4] Biomolecular Discovery and Design Research Centre, Macquarie University, Sydney, NSW 2109, Australia

* Correspondence: anwar.sunna@mq.edu.au; Tel.: +612-9850-4220

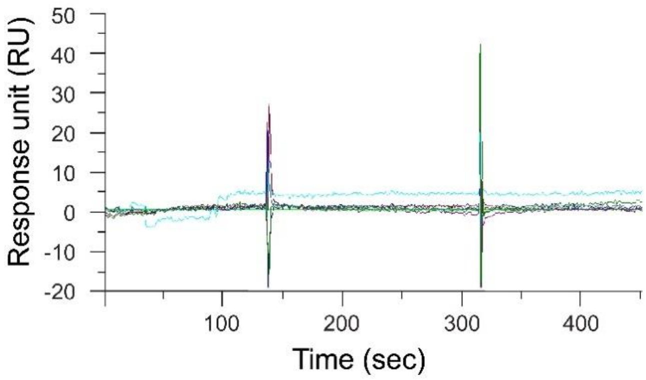

Figure S1. Global fit of 1:1 Langmuir binding model with trastuzumab for a 21-single peptide, LP1 (VKTQATSREEPPRLPSKHRPG). Black lines constitute two repeat injections of the LP1 peptide over trastuzumab immobilized on a surface. The concentrations of LP1 peptide injected were 23.1, 11.6, 5.79, 2.89 and 1.45 nM.

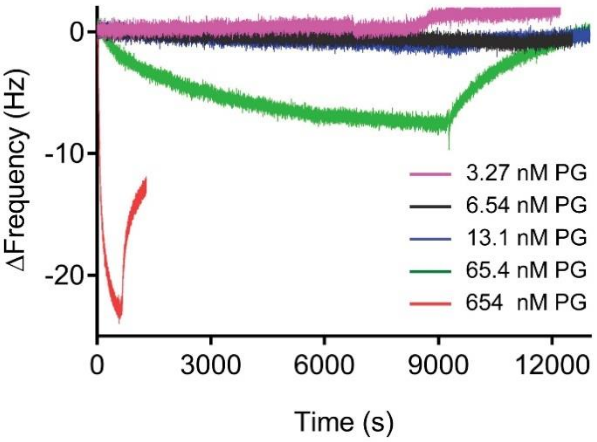

Figure S2. Frequency shift for the absorbed PG on silica coated QCM-D crystal for various concentrations (3.27-654 nM) performed on QCM-D, n=3. The measurement consists of three steps: baseline formation (PBS buffer, flow rate= 150 µL/min), adsorption (PG in PBS buffer, flow rate= 150 µL/min, flow till a saturation is achieved and then stop injection), and washing (PBS buffer, flow rate= 150 µL/min) to get rid of unbound PG. The graph showed almost all PG was dissociated after washing with PBS buffer. Three independent measurements were performed for each concentration and the graph presented in this figure is for clarification.

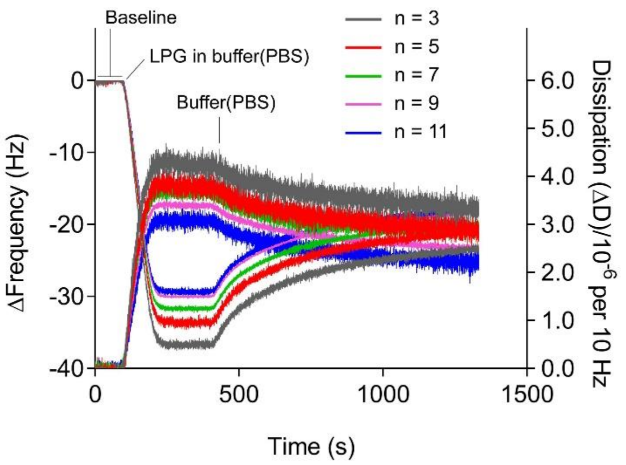

Figure S3. QCM-D signal from different overtones for LPG binding to silica coated quartz crystal. As displayed in the figure, the baseline is first established by injecting PBS buffer followed by 654 nM of LPG solution (in PBS), and finally washing with PBS buffer again. A decrease in the frequency shift is a result of LPG adsorption onto silica coated crystal. Since the signals from different overtones does not overlap and are not close to each other, with a significant dissipation respsone, the adsorbed LPG layer is viscous and multilayered.

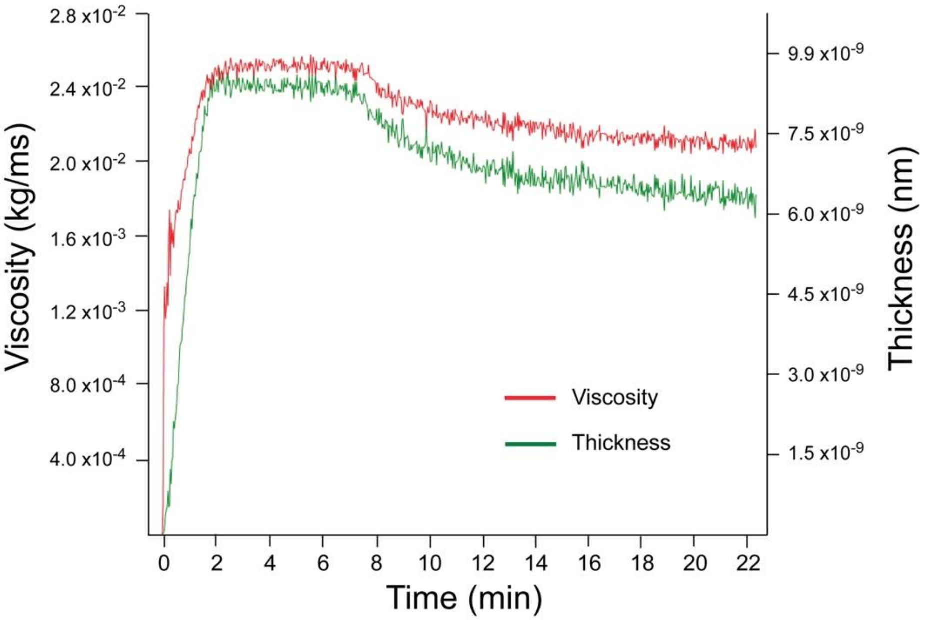

Figure S4. Overlay of the Kelvin-Voigt fitted raw data for thickness and viscosity for the adsorption of LPG to silica coated crystal, n=3 overtone. The frequency and dissipation signal for both the parameters follow each other, showing the formation of a viscous peptide layer.

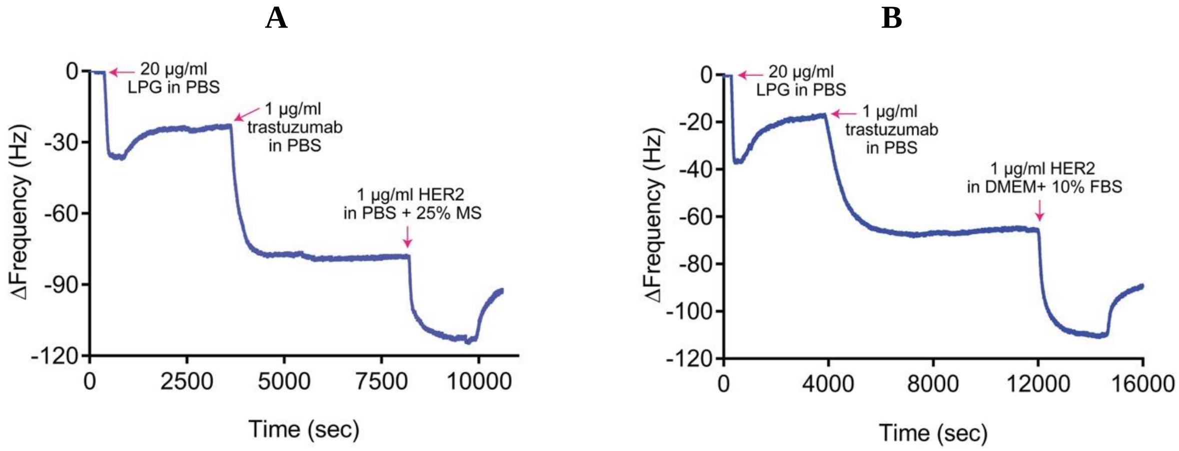

Figure S5. QCM-D response for the binding interactions of trastuzumab to LPG and subsequent detection of HER2 spiked in **(A)** PBS+25% mouse serum (MS) and **(B)** DMEM+10% fetal bovine serum (FBS). 20 µg/ml LPG (654 nM) in PBS was immobilized to silica surface and the non-specific binding was avoided using a solution of 1 mg/ml BSA in PBS. The arrows indicate the time points for the injection of LPG, trastuzumab and HER2, with PBS washing between each sample injection.

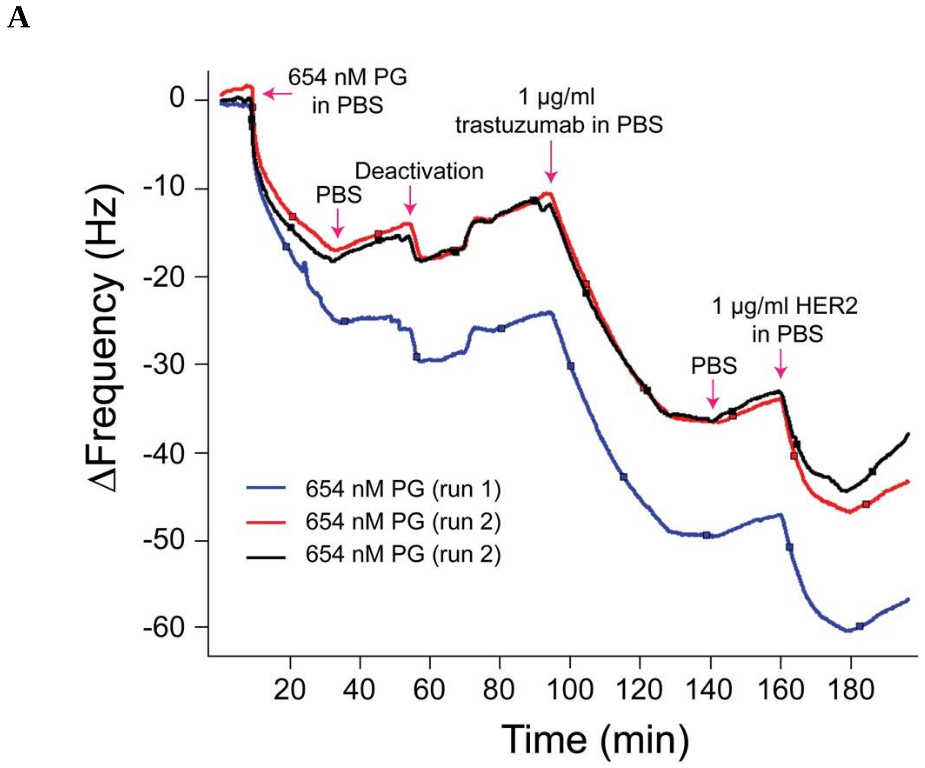

B

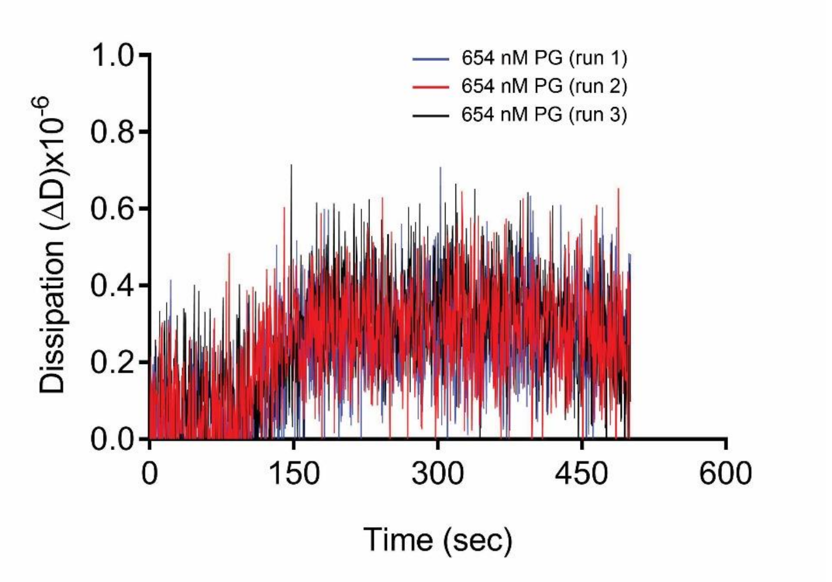

Figure S6. (A) QCM-D response for the binding interactions of trastuzumab to PG and subsequent detection of HER2 spiked in PBS. 654 nM of PG (in PBS) was immobilized to amine activated silica surface. Deactivation of the unused groups was carried by injecting 0.1 M Tris, 50 mM ethanolamine (pH 9.0), followed by blocking with 1 mg/ml BSA to avoid non-specific binding interactions. The arrows indicate the time points for the injection of PG, trastuzumab and HER2, with a PBS washing between each sample injection. **(B)** ΔD for the 3rd overtone of the chemisorbed PG on silica coated QCM-D crystals for three independent measurements at a final PG concentration of 654 nM.
